# *Escherichia coli*-derived outer membrane vesicles are genotoxic to human enterocyte-like cells

**DOI:** 10.1186/1750-9378-9-2

**Published:** 2014-01-10

**Authors:** Peter C Tyrer, Frank A Frizelle, Jacqueline I Keenan

**Affiliations:** 1 Department of Surgery, University of Otago Christchurch, PO Box 4345, Christchurch 8140, New Zealand

**Keywords:** *Escherichia coli*, Outer membrane vesicles, Genotoxicity, Colorectal cancer

## Abstract

**Background:**

Colorectal cancers are the third most common type in the world. The causes of the disease are poorly understood, but since the discovery of *Helicobacter pylori* as a causative agent of gastric cancer, attention has turned to bacteria as a possible trigger for colorectal cancer. Recently *H. pylori* outer membrane vesicles (OMVs) were revealed as potentially genotoxic which can be important first step in carcinogenesis. We therefore investigated whether OMVs from intestinal *Escherichia coli* could be genotoxic.

**Methods:**

OMVs from the avirulent DH5α strain, a pathogenic adherent-invasive *E. coli* (AIEC) and an enterohaemolytic (EHEC) strain of *E. coli* were enriched by ultracentrifugation. The effect on the growth and viability of human enterocyte-like Caco-2 cells by OMVs was determined by trypan blue exclusion, MTT and BrdU incorporation assays. The ability of OMVs to induce DNA damage was assayed by single-cell gel electrophoresis, and 8-oxo-dG and γH2Ax immunofluorescence staining. Cytopathological changes were assessed by microscopy. The induction of aneuploidy by the OMVs was measured by flow cytometry in Caco-2 and LoVo cells.

**Results:**

We found that OMVs derived were internalised by Caco-2 cells, increased cell numbers, induced double-stranded DNA breaks, recruited γH2Ax to the nucleus, initiated DNA rereplication, and produced distended multinucleate cells. DH5α and AIEC OMVs caused free radical generation as indicated by the reduction of glutathione in cells, leading to the development of mutagenic 8-oxo-dG adducts in DNA. Flow cytometry revealed that DH5α and EHEC OMVs increased aneuploidy in *p53* mutant Caco-2 cells, but not in *p53* wild type LoVo cells.

**Conclusion:**

We conclude that *E. coli* derived OMVs, whether from avirulent or pathogenic strains are potentially genotoxic.

## Background

Colorectal cancer is a significant cause of morbidity and mortality. It is the third most common cancer diagnosed amongst men and the second amongst women (663 000 and 571 000 cases each year, respectively) [1] and is particularly prevalent in Western countries, with the highest incidence in Australasia [2]. The genesis of colorectal cancer is poorly understood, but chromosomal instability is a characteristic of many sporadic colon cancers. The features of chromosomal instability include DNA damage, and failure of DNA repair, leading to aneuploidy [3].

A number of risk factors have been identified, including, but not restricted to, age, a history of inflammatory bowel disease, a family history of colorectal cancer, obesity, smoking [4,5] and alcohol use [6]. Recently, it has been proposed that intestinal bacteria, including commensals as well as pathogens, may also play a role in the aetiology of this disease [7-10]. Bacteria were first identified as a cause of cancer when *Helicobacter pylori* was shown to be involved in the development of gastric cancer [11]. Inevitably, this has led to the hypothesis that other bacteria may also initiate carcinogenesis, particularly those strains that produce toxins [12,13]. Moreover, there is mounting evidence that bacteria do not need to be invasive to affect this response. Gram-negative bacteria constitutively release outer membrane vesicles (OMVs), both *in vivo* and *in vitro*. These 50-200 nm diameter proteoliposomes consist of outer membrane and periplasmic proteins [14], lipopolysaccharide, peptidoglycans, and may contain DNA and RNA [15]. Many of the constituents of OMVs are the products of virulence genes that include toxins and/or molecules that assist in the attachment to and invasion of target host cells [16-23]. Thus, OMVs may act as long range effectors to remotely deliver toxins to host cells [24] and provide a further virulence mechanism in addition to, for example, Type IV secretion systems. We recently described that vesicles shed from the surface of a toxin-producing strain of *H. pylori* are rapidly internalised by human gastric carcinoma AGS cells [25] alter proliferation and induce oxidative stress, leading to genotoxicity that could result in chromosomal instability [26].

*Escherichia coli* are commonly found within the human intestinal tract. Indeed, most *E. coli* strains are part of the normal microflora of the gut where they contribute to food digestion. In addition, commensal strains of *E. coli* are thought to compete with potential pathogens (for example by the production of bacteriocins), so inhibiting infection [27]. In contrast, some species of *E. coli* produce toxins. Whereas these toxins cause diarrheal diseases that can lead to significant illnesses and death [28], their role as potential carcinogens is less well understood. Given the genotoxicity of *H. pylori* OMVs, we hypothesised that *E. coli* OMVs might also induce DNA damage and aneuploidy.

We used the human adenocarcinoma-derived cell line Caco-2 as a model of gut enterocytes and exposed these cells to OMVs constitutively expressed by three strains of *E. coli* that included an avirulent strain [29], an adherent-invasive *E. coli* (AIEC) strain associated with inflammatory bowel disease and colon cancer [30] and a Shiga toxin (STx)-producing, enterohaemolytic (EHEC) O157:H7 strain associated with bloody diarrheal disease and haemolytic uremic syndrome (HUS) [31]. We examined whether these OMVs were internalised by Caco-2 cells and investigated the effect that the OMVs from each of these three different strains of *E. coli* had on cell proliferation and viability. The ability of the OMVs to induce abnormal cell morphology, oxidative stress, damage DNA, and produce aneuploid cells was also assessed.

## Results

### OMVs are internalised by Caco-2 cells

**Figure 1:**
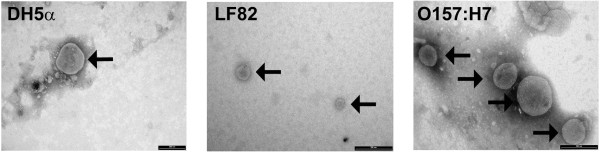
**Electron micrographs of preparations of outer membrane vesicles.***E. coli* OMVs were recovered following the overnight growth of bacteria in LB broth. OMVs are indicated by black arrows. Magnification 93 000. Size bar indicates 200 nm.

**Figure 2:**
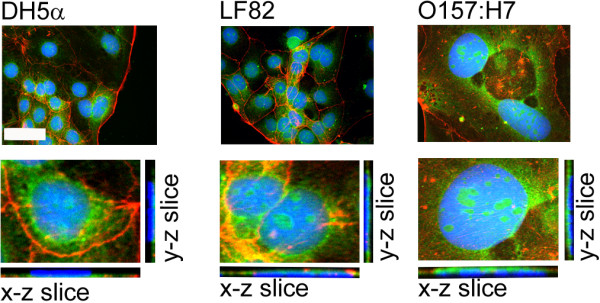
***E. coli *OMVs are internalised by and localise to the perinuclear regions of Caco-2 cells.** Fluorescent DiO–labelled OMVs (green), were added to Caco-2 cells for 4 hours before the cells were stained for actin (red) and nuclei (blue). Image stacks were taken 0.28 μm apart. The top images represent a maximum intensity z-projection constructed in ImageJ whereas the bottom images are insets of those above with their x-z and y-z orthogonal projections to show that OMVs are internalised by Caco-2 cells. Images are from a single representative experiment. Original magnification = 40×, NA1.3 objective used. Note that the figure for O157:H7 OMV treated Caco-2 cells shows an enlarged, intoxicated nucleus.

*E. coli* grown in broth have been shown to constitutively shed OMV [32]. OMVs can be enriched by ultracentrifugation with no biologically active contaminants [25]. We observed that all three strains of *E. coli* used in this study constitutively shed OMVs and these were numerous in each preparation when examined by TEM. The enriched OMVs ranged in size between 25-200 nm in diameter (arrowed in Figure 1) and these dimensions agree with previously published reports [33]. We have previously shown that in enriched OMVs preparations contaminants, such as flagella, are at least partially depleted [23,34] and in this study TEM did not reveal significant quantities of these impurities in our OMV preparations from any of the *E. coli* strains we used. Using immunofluorescence microscopy, we then determined if the OMVs produced by three *E. coli* strains used in this study are internalised by Caco-2 cells [20]. By labelling OMVs with the fluorescent, membrane-intercalating dye 3,3′-dioctadecyloxacarbocyanine perchlorate (DiO) we were able to show that vesicles entered the cells, irrespective of whether they were from attenuated laboratory or pathogenic strains of *E coli*. Moreover, orthogonal views reconstructed from image slices 0.28 μm apart, confirmed that the OMVs were located in the perinuclear regions of cells (Figure 2).

### OH157:H7 OMVs induce growth of Caco-2 cells, but viability is unaffected

**Figure 3:**
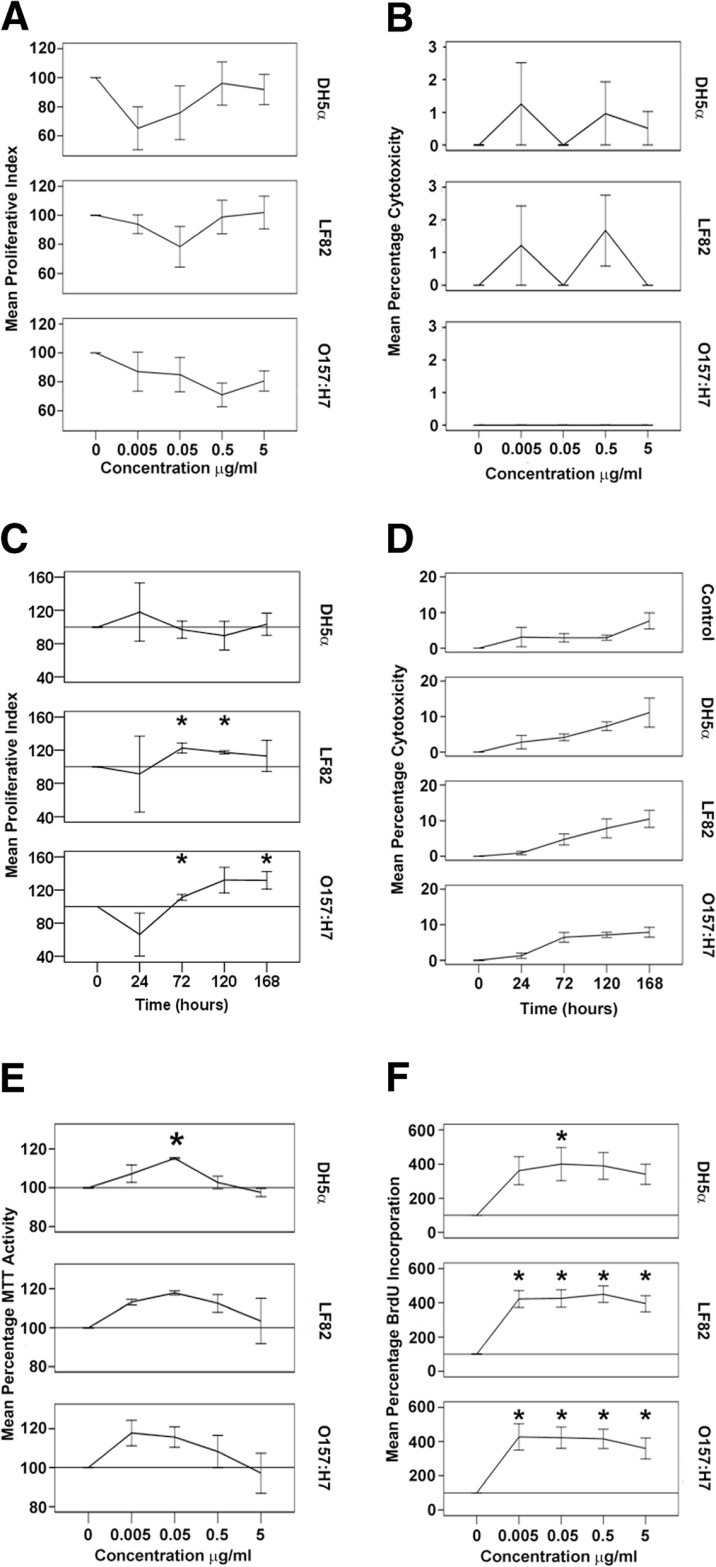
***E. coli *OMVs increase Caco-2 cell proliferation. (A)** Dose dependent proliferation indices and **(B)** percentage cytotoxicity of Caco-2 cultures after 7 days exposure to OMVs, determined by trypan blue assay. **(C)** Time dependent proliferation indices and **(D)** percentage cytotoxicity of Caco-2 cultures after 7 days exposure to OMVs, determined by trypan blue assay. **(E)** OMVs increase MTT reduction by Caco-2 cells. **(F)** OMVs increase the synthesis of DNA in Caco-2 cells as determined by BrdU incorporation assay. *, results are significantly different from untreated controls (*p* ≤ 0.05). ANOVA, followed by two-sided Dunnet’s *post hoc* test. Results are ± SEM of three independent experiments. In c, e and f, the line denotes 100% i.e. unexposed control values.

Carcinogenesis is characterised by the dysregulation of cell proliferation, resulting in uncontrolled cell growth [35], and we hypothesised that *E. coli* OMVs might increase the growth of Caco-2 cells. In dose response experiments, we determined that doses of OMVs between 0.005 and 5 μg/ml had no significant effect on the growth (Figure 3A) or viability of Caco-2 cells (Figure 3B) over 168 hours. We then monitored the effect that 5 μg/ml OMVs had on cell numbers in kinetic studies by exposing the cells to OMVs for up to 168 hours and conducting trypan blue exclusion assays every second day of culture. We observed that unstimulated control cells continued to grow for the period of the experiment, though the rate of growth began to decline slightly, as expected, as they reached confluence. Cells stimulated with OMVs derived from *E. coli* strain DH5α also continued to grow throughout the period of the experiment, but there was no significant difference in growth at any time point when the results were normalised to the untreated control cells (Figure 3C). In contrast, exposure to OMVs from the other two strains of *E. coli* accelerated the Caco-2 cell growth. For LF82 OMVs, these increases were statistically significant after 72 and again at 120 hours, (122% +/- 6, *p =* 0.021 and 117% +/- 2, *p* = 0.001, respectively, compared to control) but cell numbers declined thereafter. In the case of O157:H7 OMVs, cells numbers were significantly increased after 72 hours (111% +/- 4, *p* = 0.036) and remained increased at 168 hours (131% +/- 10, *p* = 0.039) when compared to untreated control cells (Figure 3C). In contrast, the *E. coli* OMVs had no significant cytotoxic affect on the Caco-2 cells over time (Figure 3D). Together, these results indicate that DH5α OMVs have no significant effect on cell growth. This is not surprising in the case of DH5α, which is considered to be avirulent and non-pathogenic [29]. Strain LF82 transiently increased cell growth over time and had no effect on cell cytotoxicity, as reported elsewhere [36]. OMVs derived from the more pathogenic O157:H7 strain also had no significant cytotoxic effect on Caco-2 cells. However, these OMVs were associated with a significant increase in cell growth over time, and this growth continued even when unstimulated control cells had reached confluence. These data indicate that LF 82 and O157:H7 OMVs can dysregulate cell growth.

In addition to using trypan blue to determine cell number and viability, we also used the (3-(4,5-Dimethylthiazol-2-yl)-2,5-diphenyltetrazolium bromide (MTT) [37] (Figure 3E) and 5-bromo-2′-deoxyuridine (BrdU) incorporation [38] (Figure 3F) assays, which are common high-throughput means to determine cell proliferation. Intriguingly, both assays produced results that were contrary to those obtained by use of the trypan blue exclusion assay. In the case of the MTT determinations (which infers viable cell numbers from the reduction of MTT), there was an approximately 20% increase in MTT reduction above that of controls at lower doses, that then decreased with increasing OMV concentration.

The difference between viable cell counts and the results of the BrdU incorporation assays was even more marked, with the incorporation of BrdU in OMV-treated cells measured as up to 400% of control values.

### OMVs induce glutathione reduction in Caco-2 cells

**Figure 4:**
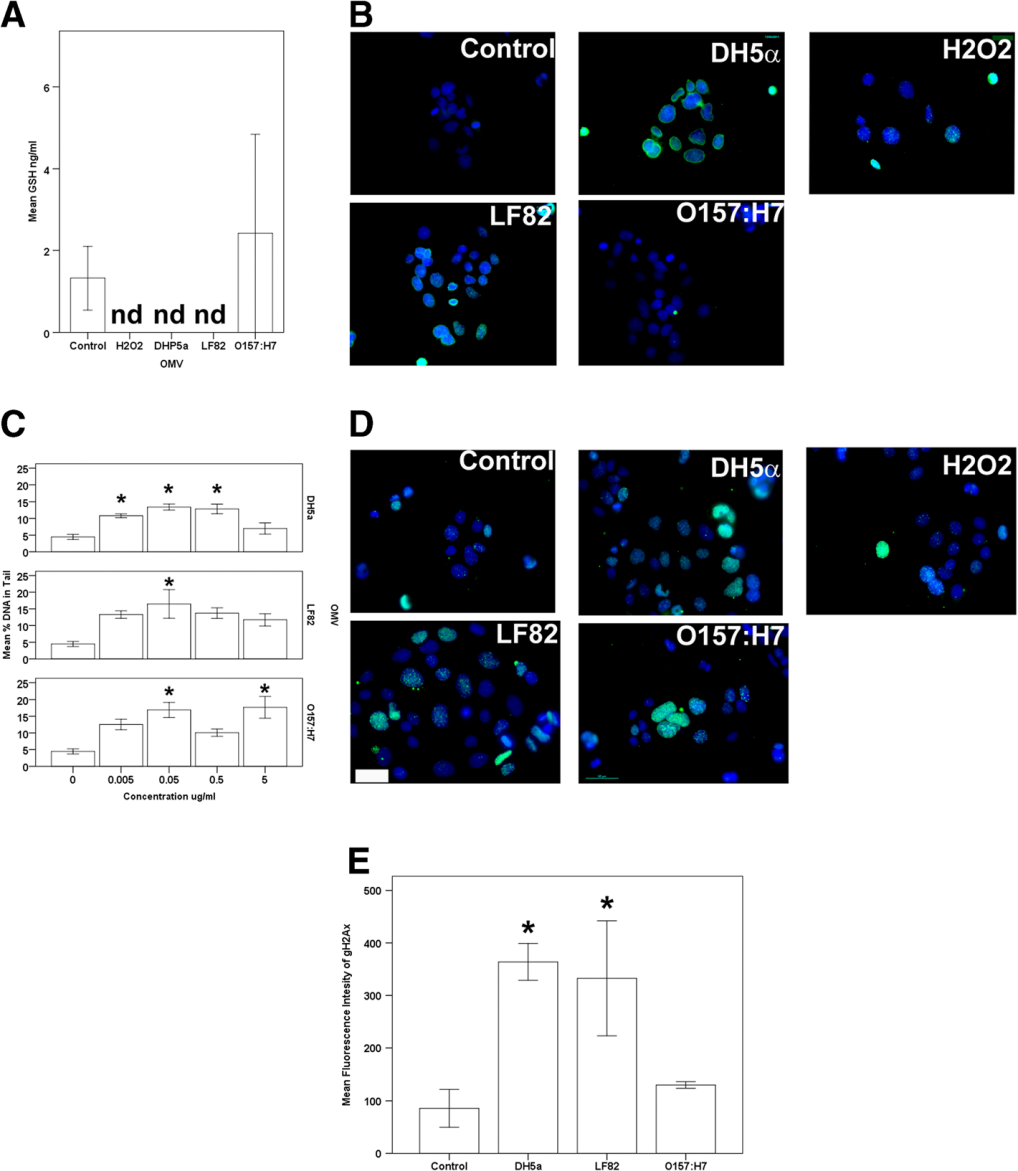
***E. coli *OMVs induce reactive oxygen species generation and DNA damage. (A)** DH5α and LF82 derived OMVs decrease cellular GSH, indicating an increase in ROS. nd denotes not detected. **(B)** DH5α and LF82 derived OMVs are associated with the formation of mutagenic 8-oxo-dG adducts (green) in Caco-2 nuclei (blue). **(C)** OMVs induce DNA double strand breaks in Caco-2 cells as measured by Comet assay. **(D)** OMVs induce the recruitment of γH2Ax histone (green) to sites of damaged nuclear DNA (blue). **(E)** Flow cytometric analysis of γH2Ax expression during the G1 phase of the cell cycle of Cacco-2 cells. **(A)**, **(C)** and **(E)**, *, results are significantly different from untreated controls (*p* ≤ 0.05). ANOVA, followed by two-sided Dunnet’s *post hoc* test. Results are ± SEM of three independent experiments. **(B)** and **(D)**, Immunofluorescence microscopy images are from a single representative experiment. Original magnification = 40×, NA1.3 objective used, scale bar indicates 50 μm.

The MTT assay is used as a technique to determine cell viability, however it does so by proxy by relying on the reduction of tetrazolium dye to formazan by NAD(P)H-dependent cellular oxidoreductase enzymes. Thus, any agent that affects the expression or activity of these enzymes can affect the assay. For example metabolic uncouplers [39,40] can falsely give rise to increased cell counts measured by this assay. Furthermore, tetrazolium can be reduced by a variety of agents without increasing cell viability, including genistein [41], ursolic acid [42], resveratrol [43], and interferons [44]. The discrepant data obtained from the MTT assay could also be due to mitochondrial dysfunction, and a consequence of this is the generation of reactive oxygen species (ROS) [45]. Thus, we hypothesised that *E.* coli OMVs might increase the generation of ROS in Caco-2 cells. There is an inverse correlation between oxidative stress and cellular glutathione (GSH) levels [46]. *H. pylori* OMVs have been shown to reduce the presence of GSH in AGS cells [26]. Accordingly, we determined the effect of *E. coli* OMVs on cellular GSH levels (Figure 4A).

The level of GSH in unstimulated Caco-2 cells was 1.4+/-0.7 nM whereas exposure to 200 μM hydrogen peroxide (a positive control to induce oxidative stress) for 24 hours reduced cellular GSH levels to below the limit of detection of the assay. Exposure to OMVs from *E. coli* strains LF82 or DH5α also had a significant effect on cellular GSH levels, again reducing them to below the limit of detection. In contrast, O157:H7 OMVs added to Caco-2 cells had no significant effect on cellular GSH levels when compared to unstimulated controls (Figure 4A), suggesting that OMVs from some but not all strains of *E. coli* have the potential to induce oxidative stress in gut epithelial cells.

### OMVs generate DNA damage in Caco-2 cells

The generation of ROS can lead to DNA damage [47,48] and the formation of mutagenic DNA adducts that include 8-oxo-7,8-dihydro-2′-deoxyguanosine (8-oxo-dG) [49]. Thus, we hypothesised that we would find evidence of 8-oxo-dG adducts within the nuclei of Caco-2 cells exposed to OMVs. Immunofluorescence microscopy was used to demonstrate that OMV exposed cells have increased staining for 8-oxo-dG, indicating a level of oxidative DNA damage not observed in untreated cells. These results suggest that OMVs from a non-pathogenic as well as a pathogenic strain of *E. coli* (DH5α and LF82, respectively) are capable of inducing mutagenic changes to genomic DNA with the potential to lead later to the initiation of carcinogenesis. In contrast, Caco-2 cells exposed to OMVs from *E. coli* strain O157:H7 did not display higher levels of 8-oxo-dG labelling (Figure 4B). This would indicate that vesicles from this strain of *E. coli* do not induce these adducts in Caco-2 cells, a finding that is consistent with the lack of oxidative stress in these cells following exposure O157:H7 OMV.

Free radicals, as well as some bacterial toxins that include the cytolethal distending toxin that is expressed by *E. coli* O157:H7 and found in their OMVs [50], can produce double stranded breaks (DSBs) in DNA [50,51]. Single-cell gel electrophoresis is a powerful technique to quantitate this damage. All three of the OMVs tested induced increases in DSBs with a peak at 0.05 μg/ml OMV for DH5α (13.42% +/- 0.92 DNA in tail, *p =* 0.001) and LF82 (16.48% +/- 4.29 DNA in tail, *p* = 0.013) that declined at higher doses compared to control (4.48% +/- 0.77 DNA in tail). In contrast there were significant increases in damage at 0.05 and 5 μg/ml O157:H7 OMVs (16.91% +/- 2.27, *p* = 0.005 and 17.68 +/- 3.27, *p* = 0.003 DNA in tail, respectively) (Figure 4C). DNA damage was also evident in Caco-2 cells exposed to 300 μM H_2_O_2_ for 24 hours (15.09% +/- 3.56 DNA in tail). Collectively, these results show that OMVs can induce DNA damage in Caco-2 cells. It is possible to speculate that he bell-shaped dose-response curves caused by DH5α and LF82 may indicate that the causes of DNA damage in response to these OMVs may be quite complex, perhaps due the induction of anti-oxidant pathways (e.g. Nrf-2) at higher doses, but this remains to be determined.

### E. coli OMVs induce DNA synthesis and repair in Caco-2 cells

DNA damage induces DNA synthesis and repair mechanisms and the large increases in BrdU incorporation described above provide some evidence for this in OMV exposed Caco-2 cells. The BrdU assay measures the incorporation of a thymidine analogue into DNA during the S-phase of the cell cycle [38]. Accordingly, this technique measures the number of actively dividing cells. However, DNA synthesis and hence BrdU incorporation, can be initiated by DNA damage, followed by repair in response to genotoxic agents [52,53]. Thus, it appears that the large increases in DNA incorporation induced by the *E. coli* OMVs could be due to DNA damage and repair. There is precedence for this. We have shown that *H. pylori* OMVs induce DNA damage to AGS gastric carcinoma cells, as determined by cytokinesis-block micronuclei assay [26].

Another marker of DNA repair is the recruitment and phosphorylation of the histone γH2Ax to the damaged site. This provides a platform for the recruitment of DNA repair enzymes that can be detected by immunofluorescence microscopy [54-56] and we hypothesised that we would detect increased staining for γH2Ax in the nuclei of Caco-2 cells exposed to OMVs. We found that occasional unstimulated cells displayed expression of γH2Ax, as indicated by the punctate green nuclear staining in fluorescence micrographs (Figure 4D). This was expected as cells often constitutively generate free radicals with resultant low level DNA damage [57]. However, following exposure to DH5α and LF82 OMVs, notably more cells displayed the green nuclear staining demonstrating that there was greater recruitment of γH2Ax to DSBs and therefore more DNA damage (Figure 4D) and/or increased initiation of DNA repair. These results were confirmed by quantification by flow cytometry of immunolabelled γH2Ax stimulated and unstimulated Caco-2 cells (Figure 4E). In unstimulated cells, the level of γH2Ax expression was 85.68 +/- 36.07 (mean fluorescence intensity in arbitary units), while DH5α OMV stimulated and LF82 OMV stimulated Caco-2 cells had statistically significant increases in γH2Ax expression (363.82 +/- 35.10, *p* = 0.029 and 332.54 +/- 109.56, 0.049 respectively, ANOVA followed by Dunnett’s 2-sided *post-hoc* test). In contrast, O157:H7 OMVs induce no significant increase in γH2Ax expression detected either by immunofluorescence microscopy or flow cytomtery. These data concur with our earlier observations regarding the increases in the incorporation of BrdU and therefore DNA synthesis.

### Caco-2 cells exposed to OMVs show significant cytopathological changes consistent with DNA rereplication

**Figure 5:**
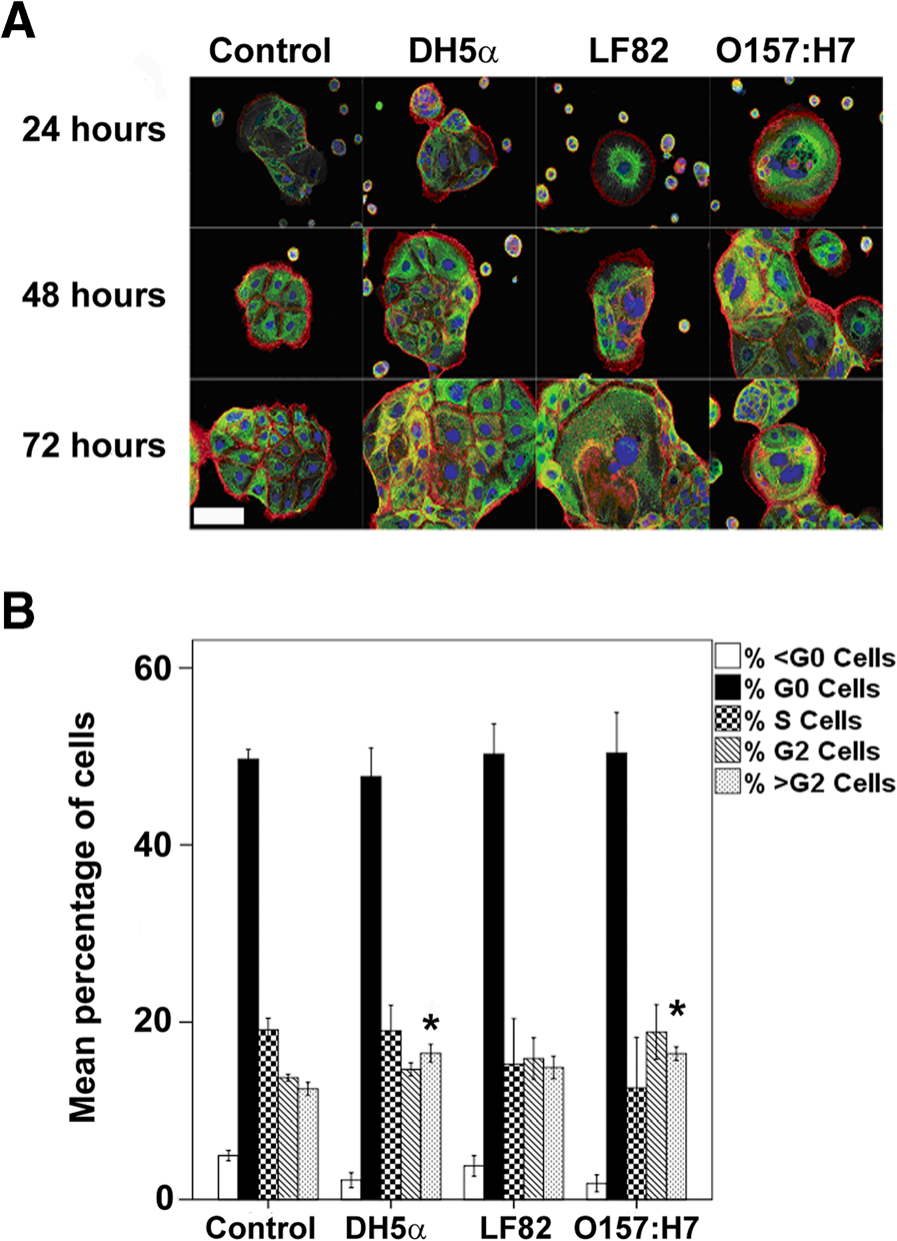
***E. coli *OMVs produce morphopathogenic changes to Caco-2 cells consistent with endoreplication and can induce aneuploidy. (A)** Immunofluorescence labelling of actin (red), cytokeratin 18 (green), and nuclei blue was used to show that OMVs cause endoreplication in Caco-2 cells over time. Images are from a single representative experiment. Original magnification = 20×, NA 0.50 objective used, scale bar indicates 50 μm. **(B)** OMVs (5 μg/ml) are associated with the generation of aneuploidy in Caco-2 cells exposed for 7 days to DH5α and O157:H7 OMVs, as measured using flow cytometry of propidium iodide stained and RNase 1 treated cells. *, results are significantly different from untreated controls (*p* ≤ 0.05). ANOVA, followed by two-sided Dunnet’s *post hoc* test. Results are ± SEM of three independent experiments.

When DNA repair fails, some cells escape cell cycle arrest and enter an abnormal mitosis. This process is described as rereplication and is characterised by significant changes in cellular morphology. Affected cells become distended, develop actin stress fibres due to improper cytokinesis, and become micro- and multinucleate [58]. We stained cells exposed to OMVs for 24, 48, and 72 hours for the cytoskeletal proteins cytokeratin-18 and actin while counterstaining with Hoechst 33342, in order to examine cellular and nuclear morphology (Figure 5A). Unstimulated cells remained largely mononucleate with nuclei of a normal size, the cells did not grow in size, and there was no evidence of actin stress fibres over the 3 day period. Also, occasional cells were observed that exhibited micronuclei. In contrast, cells exposed to OMVs displayed many of the features associated with the induction of rereplication. Cells were enlarged, becoming in some cases greater than 300 μm in diameter and additionally showed distended (50 μm diameter) nuclei. Many cells were multinucleate, had multiple micronuclei and developed actin stress fibres. Collectively, these findings suggest that exposure of cells to *E. coli.* OMVs results in the induction of rereplication.

### OMVs induce aneuploidy

After rereplication and cells become tetraploid, p53 mediated signalling induces apoptosis in a process known as mitotic catastrophe, thus deleting DNA damaged cells from the population [59,60]. This has the purpose of removing potential cancer progenitors from the population. In cells with mutations in the p53 pathway, a failure of mitotic catastrophe to delete DNA damaged cells can lead to chromosomal instability and aneuploidy in subsequent generations of daughter cells, an important mechanism of tumourigenesis [61,62]. To determine the potential of OMVs to initiate aneuploidy, we stimulated cells with 5 μg/ml OMVs for 168h. The samples were then subjected to DNA content analysis by flow cytometry (Figure 5B). Aneuploid cells can be detected by measurements of cells containing greater than > G2 DNA content. Caco-2 are a chromosomally unstable cell line and aneuploid, so we examined whether there was an increase in the percentage of cells displaying an abnormal DNA content, compared to unstimulated controls. After 168 hours, OMVs derived from O157:H7 bacteria had increased the percentage of cells that contained > G2 compared to unstimulated controls (16.45% +/- 0.76 and 12.45% +/- 0.75, respectively, *p* = 0.049), as did DH5α OMVs (16.48% +/- 1.01, *p* = 0.047). LF82 OMVs had no effect on > G2 DNA content after 168 hours (14.87+/- 1.29, *p* = 0.259). No OMVs had any statistically significant effect on the percentages of cells of sub G0, G0/1, S, or G2 DNA content.

### O157:H7 OMV induce G0/1G1 cell cycle arrest and loss of aneuploid cells in chromosomally stable and p53 positive LoVo cells

**Figure 6:**
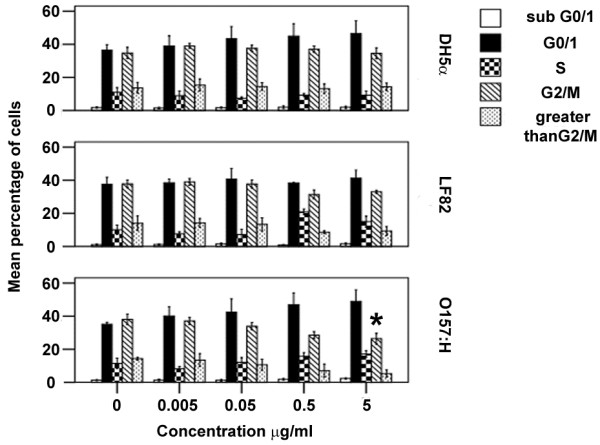
**O157:H7 *E. coli *induce cell cycle changes in LoVo cells.** LoVo cells were exposed to OMVs for 4 days, before being subjected to cell cycle analysis. O157:H7 OMV induced a dose dependent G1 arrest. *, results are significantly different from untreated controls (*p* ≤ 0.05). ANOVA, followed by two-sided Dunnet’s *post hoc* test. Results are ± SEM of three independent experiments.

Caco-2 cells are commonly used in studies of intestinal epithelia as they display many of the characteristics of enterocytes that include the expression of digestive enzymes and apical surface microvilli. However, Caco-2 cells are chromosomally unstable and therefore normally aneuploid. In addition, they also express a non-functional truncated p53. Since p53 is involved in DNA damage signalling and directs cells to either arrest at G0/1 to undergo DNA repair, or undergo apoptosis, we considered that our results from experiments using Caco-2 cells could be influenced by these two factors. We therefore examined whether *E. coli* OMVs could alter the DNA content and cell cycle of chromosomally stable and p53 wild type LoVo epithelial cells. We found that 4 day exposure to DH5α or LF82-derived OMVs had no effect on the cell cycle or aneuploidy of LoVo cells, but O157:H7 OMVs induced a dose dependent G0/1 cell cycle arrest with a significant reduction in the number of G2/M cells (38.06 +/- 3.25% and 26.41 +/- 3.27% for control and 5μg/ml O157:H7 OMVs respectively, *p* = 0.035) (Figure 6).

## Discussion

This study was performed to determine if OMVs shed from the surface of an avirulent, a putative pathogenic and an overtly pathogenic strain of *E. coli* have the potential to induce carcinogenesis in intestinal epithelial cells. By labelling the OMVs with the fluorescent dye DiO, we were able to observe the internalisation of the OMVs into Caco-2 cells. We will perform further experiments to confirm the stability of DiO-labelled OMVs at 4°C. We found that OMVs from all three strains of *E. coli* examined were internalised into Caco-2 cells, and that two of the three strains were capable of inducing oxidative stress. Intriguingly, the pathogenic strain O157:H7 was the one strain in this study that produced OMVs that did not induce oxidative stress. DNA damage, including mutagenic changes and later aneuploidy, was a consequence of exposing intestinal Caco-2 epithelial cells to *E. coli* OMVs, irrespective of strain, but OMVs from the strains LF82 and O157:H7 also significantly increased cell proliferation.

Perhaps surprisingly, OMVs from *E. coli* DH5α, an avirulent strain of *E. coli*, were capable of inducing free radical formation that was associated with genotoxic changes in cellular DNA, rereplication, and an increase in the percentage of aneuploid cells. However, results of our study are consistent with that of Koturbash *et al*[63], who showed that DH5α could induce genotoxicity in murine small intestine, liver, and spleen tissues, even after the bacteria were heat-killed. Collectively, these findings emphasise the need to examine the genotoxic potential of microorganisms currently thought to be non-pathogenic.

Adherent-invasive *E. coli* strain LF82 is associated with Crohn’s disease [36], which can lead to colon cancer later in life [64,65]. Strain LF82 and other AIEC bacteria are considered pathobionts, which are commensal bacteria that can lead to disease. Consistent with a possible role for LF82 in carcinogenesis, we found that OMVs derived from *E. coli* strain LF82 induced DNA damage, 8-oxo-dG adducts, DNA rereplication, and aneuploidy. As AIEC can colonise the gut and hence can reside in the intestine for considerable periods, the ability of their OMVs to induce ROS and DNA damage suggest that LF82 is, potentially, a significant risk for genotoxic damage within the gut.

It is not known what components of DH5α and LF82 derived OMVs could cause genotoxicity in enterocyte-like cells. We plan to perform a proteomics study to identify potential genotoxins.

Koturbash *et al*[63] also noted that heat-killed *E. coli* O157:H7 was genotoxic and our results support this. Strain O157:H7 OMVs did not induce free radical generation, as indicated by GSH assays, nor did they induce the formation of 8-oxo-dG adducts, but they did cause the formation of double strand breaks in Caco-2 cell DNA. OMVs from strain O157:H7 contain cytolethal distending toxin (CDT) [66]. CDT has significant structural and functional homology to eukaryotic Dnase I [51] and is capable of cutting through DNA strands and therefore generating double strand breaks. This results in significant cell distension, typical of rereplication, and eventually cell death through mitotic catastrophe [50]. However, instead of dying, the O157:H7 OMV-treated cells in our study continued to proliferate, had fewer cells than controls at G0/1 of the cell cycle, and a greater number of aneuploid cells. These data indicate that DNA damaged cells are not depleted from the population, remain aneuploid and could be carcinogenic. We consider that this may relate to the presence of other toxins produced by O157:H7, but this remains to be confirmed. We will conduct further proteomic analyses of O157:H7 OMVs to identify these toxins.

The effects of O157:H7 OMVs appeared to be dependent on the p53 status of the cell line used. In p53 mutant Caco-2 cells, these OMVs increased the numbers of aneuploid cells. In contrast, the number of aneuploid cells was decreased in p53 wild type LoVo cells following exposure to O157:H7 OMVs. There was a small increase in the number of sub-G0/1 cells, compared to unexposed LoVo cells, suggesting the induction of apoptosis (although this requires confirmation). Also, dose dependent G0/1, and to a lesser extent S phase cell cycle arrest was observed. O157:H7 bacteria express Shiga toxins and these are found in their OMVs [32]. Shiga-1 toxin (Stx1) induces p53 mediated cell toxicity [67]. This toxin can also cause S cell cycle arrest in HCT116 cells [68], a line that is also p53 wild type and chromosomally stable. Our results, which are consistent with these findings, indicate that O157:H7 OMV can induce cell cycle arrest, leading to eventual cell death and removal of aneuploid cells in p53 wild type cells. We will perform additional experiments that involve the knocking in of functional p53 into Caco-2 cells and knocking out of p53 in LoVo cells to further confirm our findings.

Recently, the product of the *pks* pathogenicity island colibactin has been found to be genotoxic [69] and prevalent in many pathogenic and commensal B2 *E. coli*[70]*.* It is of note that none of the three strains of *E. coli* examined in this study possess this pathogenicity island [71-73], and therefore the genotoxicity of their OMVs cannot be attributed to colibactin.

Genotoxicity and alterations in cell growth are significant contributors to carcinogenesis. We have shown that each of three *E. coli* OMVs tested in this study were genotoxic and two were capable of inducing alterations in the rate of cell proliferation in a time-dependent manner. However, these data do not indisputably show that these OMVs are carcinogenic. Further studies, for example in the APC min -/- mouse model [74], are required to confirm this.

## Conclusion

In summary, we have shown that *E. coli*-derived OMVs are readily internalised into Caco-2 cells, where they are potentially capable of inducing oxidative stress, leading to DNA damage, rereplication and aneuploidy in susceptible cells. In addition, O157:H7 derived OMVs generated aneuploid cells typical of those with chromosomal instability. Thus, we consider *E. coli* derived OMVs to be genotoxic.

## Materials and methods

### Materials

DMEM, MEM non-essential amino acids, 100× penicillin/streptomycin, foetal bovine serum, Dulbecco’s phosphate-buffered saline (D-PBS), 0.25% w/v trypsin-EDTA, Vybrant™ DiO (3,3′-dioctadecyloxacarbocyanine perchlorate), Hoechst 33342, TRITC-phalloidin, SlowFade antifade reagent, and Alexa 488 and Alexa 594 conjugates were all obtained from Life Technologies (Mulgrave, Victoria, Australia). Vivaspin 500 ultrafiltration were from Sartorius Stedim Biotech GmbH (Goettingen, Germany). Anti-8-oxo-2′-deoxyguanosine (8-oxo-dG) antibody was bought from Trevigen, Inc. (Gaithersburg, MD, United States). Glutathione Assay Kit was purchased from Cayman Biosciences (Sapphire Biosciences, Auckland, New Zealand). Cell Proliferation ELISA, BrdU (colormetric) was obtained from Roche Diagnostics GmbH (Mannheim, Germany). All other reagents were bought from Sigma (Auckland, New Zealand).

### Bacterial strains

Three *E. coli* strains were used in this study. A non-pathogenic E. coli laboratory strain DH5α [29], adherent-invasive E. coli (AIEC) strain LF82 [75] and enterohaemolytic E. coli (EHEC) (CDC strain G5244) serotype O157:H7 [New Zealand Reference Culture Collection] [31]. LF82 was kindly provided by Dr. Darfeuille-Michaud (Université d'Auvergne, Clermont-Ferrand, France).

#### Harvesting and labeling of OMVs

Outer membrane vesicles were harvested as previously described [25]. Briefly, *E. coli* were grown overnight in Luria Bertani (LB) broth at 37°C with constant rotation (120 rpm). Bacteria were removed by two centrifugations (10 000 × *g*, 15 minutes, 4°C) and the final supernatants ultracentrifuged (150 000 × *g*, 3 hours, 4°C) to recover OMVs. The resultant OMV pellet was washed twice times in 0.15 M pH 8.2 Tris-buffered saline (TBS) and filtered through a 0.45 μm syringe filter (to remove any remaining bacteria). Vesicles to be labeled were washed once after recovery then, to standardize labeling, were suspended in 1 ml TBS/50 mg OMV pellet. OMVs were labeled with 1% Vybrant™ DiO by incubation for 20 minutes at 37°C. Free dye was removed by three washes with 300 000 Da cutoff Vivaspin 500 ultrafiltration units. Briefly, OMVs were added to the units with 500 μl of TBS, and then centrifuged at 14 000× *g* for 25 minutes. The retentate (containing the OMVs) was diluted with 500 μl of TBS and the process repeated twice more. After before being assayed for protein content using a modification of the Lowry assay [76], unlabeled OMVs were stored at -20°C until required whereas labelled OMVs were stored at 4°C for up to six weeks.

#### Transmission electron microscopy of OMVs

Transmission electron microscopy (TEM) was used to determine the quality of the OMV preparation. Samples of OMVs were placed on hydrophilic EM grids and stained with 1% v/v phosphotungstic acid, pH 6.8 and viewed with a Philips CM100 transmission electron microscope (Philips/FEI Eindhoven, The Netherlands) operating at 100kV. Images were captured using a MegaView 3 camera (Soft Imaging System GmbH, Münster, Germany).

### Cell culture

The Caco-2 and LoVo cells were routinely cultured in Dulbecco’s Modified Eagles Medium with Glutamax I, 4500 mg/l glucose and pyridoxal, supplemented with 10% v/v foetal calf serum, 1% v/v MEM non-essential amino acids, 50 units/ml penicillin and 50 μg/ml streptomycin. The cells were incubated at 37°C, in 5% CO_2_ and Caco-2 cells passaged 1:10 every 7 days, while LoVo cells were passaged 1:10 every 5 days. Cells (1 × 10^5^ per ml) were routinely plated and cultured for 24 hours (to allow cells to attach) before the addition of OMVs.

#### Uptake of OMVs by Caco-2 cells

Caco-2 cells plated overnight on ethanol sterilised poly-lysine coated coverslips in 6-well tissue culture plates were then incubated with labelled OMVs (5 μg/ml) for 24 hours. The cells were washed three times with D-PBS, and then fixed in 10% v/v neutral buffered formalin for 15 minutes, before permeabilisation with 0.1% v/v Triton X-100 in D-PBS for 15 minutes. Non-specific binding was blocked with 5% w/v bovine serum albumin in D-PBS for 1hour then stained with TRITC-phalloidin for 1 hour, before counterstaining with 1 μg/ml Hoescht 33342 for 15 minutes. The coverslips were then mounted in ProLong Gold mounting medium. The slides were imaged with a Zeiss Imager Z1 microscope, 40× NA 1.3 objective, Zeiss Apotome structured illumination system and Zeiss MRc camera. Image stacks were taken at 0.28 μm intervals. Images were adjusted for presentation (brightness, contrast and 3-dimensional reconstruction) with NIH ImageJ.

#### Effect of OMVs on cell viability and proliferation

The ability of the OMVs to affect the long-term viability and proliferation of Caco-2 cells was assessed by Trypan blue exclusion. Caco-2 cells plated into 24-well plates were exposed to 5 μg/ml OMVs for up to 168 hours. At two day intervals, the cells were trypsinised, stained with 0.25% w/v Trypan blue, and counted with a haemocytometer. The Mean Proliferative Index (MPI) at any given point was calculated as:

$$\begin{array}{l} \mathrm{MPI}=(\mathrm{Number}\;\mathrm{of}\;\mathrm{cells}\;\mathrm{treated}\;\mathrm{with}\;\mathrm{OMVs}\;\mathrm{in}\;\mathrm{each}\;\mathrm{well}/\\ \;\;\mathrm{Number}\;\mathrm{of}\;\mathrm{control}\;\mathrm{cells}\;\mathrm{in}\;\mathrm{each}\;\mathrm{well})\times 100. \end{array}$$

The dose-dependent effects of OMVs on the proliferation of Caco-2 cells cultured for shorter periods of time were also determined using the MTT and BrdU assays. The MTT assay is based on the mitochondrial reduction of tetrazolium to formazan, a reaction that is considered directly proportional to viable cell numbers. In contrast, the BrdU assay determines incorporation of the thymidine analogue BrdU into DNA during S-phase of the cell cycle to measure proliferation. Caco-2 cells (1 × 10^4^) were plated (in 100 μl of media) into the wells of a 96-well plate and incubated for 24 hours at 37°C before the addition of *E. coli* OMVs at concentrations between 0.5 ng/ml and 5 μg/ml. After incubation at 37°C for a further 24 hours, 20 μl of 2.5 mg/ml MTT was added to each well and the plate incubated for 5 hours at 37°C. The media was then discarded and 100 μl isopropyl alcohol containing 0.04 M HCl added to each wells. The absorbance of the wells was read at 490 nm with correction at 650 nm. For the BrdU assay, cells were plated and treated as described for the MTT assay and then the degree of BrdU incorporation was determined by Roche Cell Proliferation ELISA, BrdU (Colorimetric), according to manufacturer’s instructions.

#### Fluorescence microscopy of the cytopathological effects of OMVs

Caco-2 cells (100 000) plated overnight on ethanol sterilised coverslips in tissue culture plates were incubated with labelled OMVs (5 μg/ml) for the times indicated. The cells were fixed with 10% neutral buffered formalin for 15 minutes, and then permeabilised with 0.1% v/v Triton X-100 for 15 minutes. Potential non-specific binding sites were blocked by incubating the coverslips for 1 hour 37°C in 5% w/v bovine serum albumin in D-PBS (blocking buffer). The cells were probed with 1 μg/ml mouse anti-cytokeratin antibody in blocking buffer for 1 hour at 37°C, washed 3 times with D-PBS, then probed again with 1/500 dilution of anti-mouse Alexa 488 conjugate antibody in blocking buffer for 1 hour at 37°C. The cytoskeletal protein actin was stained with TRITC-phalloidin for 1 hour. After washing, the cells were counterstained with 1 μg/ml Hoechst 33342, mounted with SlowFade mounting media and photographed.

#### Measurement of oxidatative stress by glutathione assay

The potential of OMVs to induce oxidative stress in Caco-2 cells was determined by measurement of glutathione (GSH). Caco-2 cells (100 000) were plated overnight in 24-well tissue culture plates and then exposed to 5 μg/ml of OMVs, or 300 μM H_2_O_2_ (positive control) for 24 hours. The cells were lysed by freeze thawing from -80°C three times before assay with Cayman Glutathione Assay Kit, used according to manufacturer’s instructions.

#### Immunofluorescence microscopy of mutagenic 8-oxo-dG adducts in cells exposed to OMVs

The generation of free radicals in cells can lead to the development of mutagenic 8-oxo-dG adducts in DNA. Immunofluorescence microscopy was used to identify the presence of these lesions in OMV exposed Caco-2 cells. For the detection of 8-oxo-dG in cell nuclei, we followed the primary antibody manufacturer’s instructions. Briefly, the cells were washed 3 times with D-PBS, and then fixed with -30°C 100% v/v ethanol for 20 minutes. After treatment with 0.05N HCl for 5 minutes on ice followed by three washes in D-PBS, the cells were exposed to 100 μg/ml RNAse in 150 mM NaCl, 15 mM sodium citrate for 1 hour at 37°C before being washed sequentially in D-PBS, 35%, 50%, and 75% ethanol for 3 minutes each. DNA was denatured by addition of 0.15 N NaOH in 70% v/v ethanol for 4 minutes, followed by two washes in D-PBS after which the cells were exposed sequentially to 70% ethanol containing 4% v/v formaldehyde, 50% and 35% ethanol, then D-PBS for 2 minutes each. After incubation in 5 μg/ml proteinase K in 20 mM Tris and 1 mM EDTA, pH 7.5 (10 minutes each), the cells were washed 3 times in D-PBS before immunolabeling (as above) with anti-8-oxo-dG antibody and Alexa 488 and counterstaining with Hoechst 33342.

#### Alkaline single-cell gel electrophoresis (Comet assay) of DNA damage in OMV-exposed Caco-2 cells

DNA damage is a feature of carcinogenesis. We used the alkaline single-cell gel electrophoresis (Comet) assay to determine the degree of double-strand DNA breaks (DSBs) induced by OMVs and hence their genotoxicity. Clear glass slides were coated twice with 1% w/v normal melting point agarose then dried in a 37°C oven. Adherent Caco-2 cells exposed to either OMVs (concentrations between 0.005 and 5 μg/ml) or 300 μM H_2_O_2_ (positive control) for 24 hours were trypsinised, washed twice in D-PBS and added to 70 μl 1% low melting point agarose, which was then spread on the coated slides. The agarose-suspended cells were covered with a 22 × 22 mm No1 coverslip and the slides left at 4°C for approximately 30 minutes to allow the agarose the set before the cells were lysed overnight at 4°C in lysis buffer (2.5 M NaCl, 0.1 M EDTA, 10 mM Tris, 1% v/v Triton X-100, pH 10). The slides were transferred to a BioRad horizontal electrophoresis tank containing electrophoresis buffer (0.3 M NaOH, 1 mM EDTA) and left for 40 minutes to equilibrate before being electrophoresed for 25 minutes at 0.5V/cm. After three washes in neutralisation buffer (0.4M Tris pH 7.5) the cells were stained with 0.5 μg/ml Hoechst 33342. The slides were imaged with a Zeiss Imager Z1 with 20× NA 0.5 objective and Zeiss MRc camera. The percentage of DNA in each comet tail (linearly proportional to DNA break frequency) of at least 100 cells per sample was measured with Comet Score IV software (TriTek Corp, VA, USA).

#### Detection of the DNA damage response histone γH2Ax in cell nuclei

The localisation of phosphorylated histone γH2Ax, which is recruited to sites of DNA damage, was determined in OMV-exposed Caco-2 cells using immunofluorescence microscopy. The cells were treated as described for detection of the cytopathological changes, except mouse-anti-γH2Ax antibody (1 μg/ml) replaced the anti-cytokeratin antibody and the phalloidin staining was omitted.

For quantification of the presence of γH2Ax in the nuclei of OMV exposed cells flow cytometry was used. As γH2Ax can be expressed during the S and G2/M phases of the cell cycle due to DNA replication, only γH2Ax found in G1 was used for quantifiaction as this represents expression due to DNA damage [77]. Caco-2 cells were plated into 24-well tissue culture plates were exposed to 5 μg/ml OMVs for 7 days, then washed in culture medium to remove unbound OMVs. The cells were trypsinised, then fixed in ice-cold ethanol, and stored at -30°C until analysis. The fixed cells were washed three times with D-PBS, the treated with 200 μg/ml RNAse, and stained with 10 μg/ml propidium iodide for at least 30 minutes at room temperature. The cells were then blocked in 5% BSA for 1 hour at 37°C, then incubated in 1 μg/ml anti-gH2Ax antibody for 1 hour at 37C. After washing three times in PBS, 1/500 anti-mouse Alexa 488 was added to the samples for 1 hour at 37°C. The cells were washed then analysed using a Beckman Coulter Cytomics FC500 flow cytometer. γH2Ax positive cells were measured from data gated to the G1 phase of the cells cycle Weasel v3.0 software (Walter and Elizabeth Hall Institute, Melbourne, Australia).

#### Flow cytometry of Caco-2 and LoVo cell DNA content

A feature of carcinogenesis is the development of chromosomal instability, leading to the generation of cells with abnormally high DNA content (aneuploidy). Flow cytometry was used to assay these changes in OMV exposed cells. As Caco-2 cells are karyotypically unstable, we also used the more stable LoVo cells to investigate the effect of *E. coli* OMVs on cellular DNA content. Cells (Caco-2 or LoVo, 100 000) were plated into 24-well tissue culture plates were exposed to 5 μg/ml OMVs for 7 days, then washed in culture medium to remove unbound OMVs. The cells were trypsinised, then fixed in ice-cold ethanol, and stored at -30°C until analysis. The fixed cells were washed three times with D-PBS, the treated with 200 μg/ml RNAse, and stained with 10 μg/ml propidium iodide for at least 30 minutes at room temperature. Fluorescence measurements were made using a Beckman Coulter Cytomics FC500 flow cytometer. Cell cycle analysis and the analysis of aneuploidy (cells with greater than G2/M content) was performed with the cell cycle measurement tools of Weasel v3.0 software that deconvolves flow histograms. Gaussian models were fitted to the histogram data to obtain the percentage of cells with sub G1, G0/1, S, G2/M and > G2/M DNA content. Typically, the results were obtained after convergence at 700 iterations.

### Statistical analysis

Data are expressed as mean +/- standard error of the mean (SEM) of three independent experiments. Student’s *t*-tests and analysis of variance (ANOVA) with Dunnett’s *post hoc* test was used as tests of statistical significance, as indicated in the Results. *P* values less than or equal to 0.05 were considered to be significant. Analyses were performed with IBM SPSS versions 17, 19 and 22.

## Competing interests

We declare that we have no competing interests.

## Authors’ contributions

PT cultured the cells, performed and designed the experiments, and conducted the statistical analyses. In addition he wrote the manuscript. FF contributed to the experimental design and manuscript. JK grew bacteria and prepared the OMVs, designed the experiments and contributed to the manuscript. All authors read and approved the final manuscript.
